# Notes and Notelets

**Published:** 1909-09

**Authors:** 


### Notes and Notelets.

---

Dr. J. W. Kenney, of San Antonio, is attending clinics in Berlin and Vienna.

---

Dr. Vard H. Hulen, formerly of Houston but who went to San Francisco and for some years filled the chair of opthalmology in the University of California, has returned to Houston to remain.

He has recently returned from an extensive tour of the European clinics.

---

Dr. J. M. Frazier, of Belton, after ten weeks at Rochester at the Mayo clinics, is taking p. g. at Chicago.

---

If any fellow says, "Is it hot enough for you, Doctor?" kill him. The courts have held that it is justifiable. Fletcher's favorite poet is Chawsir.

---

Married at Austin August 4th, Dr. G. C. Mathis, of Manor, Texas, to Bessie Caperton, an Austin beauty and a society belle. Congrats, Doctor.

---

"Jimmie is a good boy," said the father, "but he will steal." I read the following in the papers today: "He was a model citizen, but occasionally drank too much."

---

Crothers, the famous editor of the *Journal of Inebriety,* says that even one ounce of alcohol, daily, will produce degenerative changes in the liver and kidneys that will shorten life.

---

"The more I see of men the better I like dogs," said the temperance man. "In what respect are dogs better than men?" asked he of the breath spiritual. "They do not get drunk and beat their wives and children, for one thing."

---

For acute coryza,—cold in the head, sneezing, lachrymation—"distemper," nazeptic wool (S. & D.) is a specific. It is absorbent cotton saturated with phenol, menthol, etc., and dried. A pledgit placed lightly in each nostril at bedtime will give entire relief. I speak from recent experience.—Ed. Red Back.

---

No diploma required now. Any old thing can join the A. M. A. (for $5.00). Dr. Simmons, First Lord and Poo Bah, in his alleged reply to charges, said,—in describing how he became a regular (and secured a diploma from Rush Medical in twenty-two days' actual attendance): "If conditions now existing had then obtained, this would not have been necessary."

